# Comparison of eating disorders symptoms and body image between individual and team sport adolescent athletes during the COVID-19 pandemic

**DOI:** 10.1186/s40337-022-00644-4

**Published:** 2022-08-12

**Authors:** Morteza Homayounnia Firoozjah, Shahnaz Shahrbanian, Alireza Homayouni, Heather Hower

**Affiliations:** 1 Department of Physical Education, Farhangian University, Tarbiat Moalem Street, Tehran, Iran; 2 Department of Sport Science, Faculty of Humanities, Tarbiat Modares University, Jalal Al Ahmad Street, Tehran, Iran; 3 Department of Psychology, Bandargaz Branch, Islamic Azad University, Bandar-e-Gaz Street, Bandargaz, Iran; 4 Department of Psychiatry, Eating Disorders Center for Treatment and Research, University of California at San Diego School of Medicine, 4510 Executive Drive, San Diego, CA 92121 USA; 5 Department of Health Services, Policy, and Practice, Hassenfeld Child Innovation Institute, Brown University School of Public Health, 121 South Main Street, Providence, RI 02903 USA

**Keywords:** Eating disorders, Body image, Adolescent athletes, COVID-19 pandemic

## Abstract

**Background:**

COVID-19 has significantly disrupted the routines of school sports for adolescent athletes, which can affect their usual eating behaviors and body image. Specific pressures of individual sports (which tend to emphasize “leanness” as a means to improving performance), versus team sports (which tend to not require “leanness” for an athlete to be competitive), may further increase the risk of disordered eating (DE), eating disorders (ED), and distorted body image. An additional factor to consider is the gender of the athletes, with participation in “lean” sports associated with increased DE and body dissatisfaction for male, but not female, athletes.

**Methods:**

Participants of the study included 124 Iranian male adolescent athletes residing in Mazandaran province (one of the most affected areas of Iran during COVID-19), who played in 1 of 6 sports (3 individual, 3 team). ED symptoms were assessed by the Eating Attitudes Test-26 (EAT-26), and body image was assessed by the Body-Esteem Scale for Adolescents and Adults (BESAA).

**Results:**

The individual athlete group (n = 62) had significantly higher EAT-26 subscale scores for Bulimia and Food Preoccupation (*p* = 0.019), as well as significantly higher BESAA subscale scores for Appearance (*p* = 0.001), Weight (*p* = 0.001), and Attribution (*p* = 0.001), compared to the team athlete group (n = 62). However, there were no significant differences between the two athlete groups on the EAT-26 Dieting and Oral Control subscales.

**Conclusions:**

COVID-19 presents specialized issues for adolescent athletes, particularly those at risk for, or experiencing, DE, ED, and distorted body image. While individual athletes had significantly higher scores than team athletes on most subscales, there were no differences between groups on subscales of dieting and oral control. Overall, the findings highlight the need for sport psychologists, coaches, and other sports professionals working with male adolescent athletes (individual and team) to monitor DE, ED, and distorted body image during COVID-19, in order to provide early intervention, and mitigate the risk of long-term consequences.

**Plain English Summary:**

COVID-19 has significantly disrupted the routines of school sports for adolescent athletes, which can affect their usual eating behaviors and body image. Specific pressures of individual sports (which tend to emphasize “leanness” as a means to improving performance), versus team sports (which tend to not require “leanness” for an athlete to be competitive), may further increase the risk of disordered eating (DE), eating disorders (ED), and distorted body image. An additional factor to consider is the gender of the athletes, with participation in “lean” sports associated with increased DE and body dissatisfaction for male, but not female, athletes. Participants of the study included 124 Iranian male adolescent athletes in Mazandaran province (one of the most affected areas of Iran during COVID-19), who played in 1 of 6 sports (3 individual, 3 team). The individual athlete group (62 participants) had significantly higher scores on measures of ED (Bulimia, Food Preoccupation), and body image (Appearance, Weight, Attribution), versus the team athlete group (62 participants). However, there were no significant differences between groups on other measures of ED (Dieting, Oral Control). Findings highlight the need for sport professionals working with adolescent athletes to monitor DE and body image during COVID-19 for early intervention.

**Supplementary Information:**

The online version contains supplementary material available at 10.1186/s40337-022-00644-4.

## Background

### COVID-19 general population lowered physical activity and increased psychological distress

The global outbreak of COVID-19, from March 2020 to date, has resulted in closure of multiple recreational facilities (e.g., gyms) around the world. Many individuals are not able to participate in their regular individual or group physical activities. According to the World Health Organization (WHO), under such conditions, many people tend to be less physically active, have longer screen time, irregular sleep patterns, as well as reduced quality of diet, potentially resulting in weight gain, and loss of physical fitness, which can have a distinct impact on athletes [1]. COVID-19 has also been associated with significantly higher levels of psychological distress, including elevated rates of anxiety and depression, which can have a negative effect on people’s functioning in different domains, overall well-being, identity, and quality of life [2]. Indeed, there was an early (April 2020) call to action for multidisciplinary, collaborative, mental health research conducted within populations whose lives are impacted by the pandemic [3].

### COVID-19 adolescent athlete lowered physical activity and increased psychological distress

Research on the specific population of adolescent athletes with lowered physical activity (e.g., restricted exercise routines) and increased psychological distress (e.g., exacerbated eating and body image concerns) during COVID-19 is very limited, given the narrow focus of this study group, and the acute onset of the pandemic. With this caveat in mind, we can learn from existing studies on aspects of this population, and dependent variables of interest, in order to compare and contrast findings with the current study. Overall, the existing literature thus far indicates that adolescent athletes have reported decreased physical activity, increased depression and anxiety symptoms, and lower quality of life, during the COVID-19 pandemic [4, 5].

Near the beginning of the pandemic (May 2020), McGuine et al. compared changes in health measures of adolescent athletes in a cohort of participants prior to the pandemic (2015–2018) to a cohort of participants during the pandemic (May 2020) [4]. They reported that the adolescent athletes’ scores for physical activity, depression symptoms, and quality of life were lower during COVID-19, compared to years earlier [4]. The authors reaffirm that quantifying these types of changes in subsequent research studies will allow health care providers to implement strategies to improve the health of adolescent athletes during the pandemic [6].

Denerel and colleagues assessed the effects of long-duration home isolation linked to the pandemic on the mental health of adolescent athletes in June 2021 [5]. In a study that included 940 team athletes, 274 individual athletes, and 131 nonathlete controls (both boys and girls, age range 12–17 years), they reported that 88% did not meet the physical activity recommendations for children and adolescents, and 42.8% felt depressed. Among athletes, depressive and anxiety symptoms were lower compared with nonathlete controls (*p* < 0.01). Posttraumatic stress symptoms were lower among athletes than nonathlete controls for girls (team vs. control, *p* = 0.006; individual vs. control, *p* = 0.002) but similar for boys (*p* > 0.05). The depression (*p* = 0.518), state (*p* = 0.866), and trait anxiety (*p* = 0.507) symptoms were similar between team athletes and individual athletes. The authors reaffirmed that, though adolescent athletes’ depression, anxiety, and posttraumatic stress symptoms were significantly lower than nonathlete controls, athletes also had high depression levels, indicating the need to take precautions to protect the psychological health of athletes in the pandemic [5].

### COVID-19 disruption of adolescent athlete routines

COVID-19 has significantly disrupted the routines of school and sports for adolescents (e.g., in-person training activities, organized sporting events), which can affect their usual eating behaviors, body image, and exercise patterns [4, 7–12]. The increased focus on body weight during adolescence can lead to disordered eating (DE), eating disorders (ED), and body image concerns [13]. Isolation, including during the COVID-19 lockdown, can additionally challenge an adolescent athlete’s ability to maintain their usual healthy nutrition and performance levels [14]. Athletes, used to a high caloric intake, may maintain the same eating routines, despite less exercise, and evolving mood-related drivers of comfort eating occurring with boredom and stress [8]. Some of the beneficial physical adaptations in the body observed as a result of a regimented training program (e.g., increases in blood and blood plasma volume, cardiac output and stroke volume during maximal efforts, muscular hypertrophy) [15] have been shown to reverse with deconditioning (e.g., extended periods of lowered or inactivity) [16]. Thus, adolescent athletes may be particularly vulnerable to these concerns during this extended COVID-19 lockdown [3–12].

### The DE to ED spectrum

The DE to ED spectrum ranges from the DE subclinical state, characterized with less cognitive and behavioral symptomatology [9, 17], to the clinical ED diagnoses, as defined by the Diagnostic and Statistical Manual of Mental Disorders-5 (DSM-5) [18]. The DSM-5 includes the primary diagnoses of Anorexia Nervosa (AN), characterized by restriction and low weight, and Bulimia Nervosa (BN), characterized by recurrent episodes of binging and purging. ED are often contributed to by a combination of genetic predisposition (family history) and environmental triggers (e.g., sport-specific pressures, COVD-19 stress). ED are associated with certain risk factors which may be especially developmentally relevant for adolescent athletes. For most ED, the age of onset is usually in adolescence, and the consequential effects on the developing body can significantly interfere with an adolescent’s athletic performance. Indeed, research indicates that individuals with a family history of AN, who have childhood anxiety traits, and pursue avocations that encourage thinness (e.g., elite athletes), are at an increased risk for developing AN in adolescence [18]. Similarly, individuals with a family history of BN, who have childhood obesity, subsequently attempted to restrict food and/or overexercise (e.g., through sports), but ended up binging later, are at increased risk for developing BN in adolescence [18].

### Body image

A cross-cutting symptom of the above primary types of ED is the concern about body image, which is the internal representation of an individual's external appearance (e.g., self-evaluations of one’s physical appearance) [19]. Given that appearance is an integral part of one’s identity, and it plays a role in social situations, body image is an important aspect of life for most people, and particularly for those individuals who are at risk for, or are experiencing, an ED [20].

Formative research on body image in adolescent male athletes has indicated that there are significant differences between the types of sports that athletes participate in, and their related body image concerns [21]. A study by Parks and Read [21] compared adolescent male football players (team sport) (n = 44) and cross-country runners (individual sport) (n = 30) on measures of weight concerns, and perceived/ideal body shape/size. They noted that football players reported a more positive body image, while cross-country runners indicated a greater degree of body dissatisfaction, and concern for weight control [21]. The authors concluded that the cross-country runners’ group should be identified for increased need in health education [21].

Emerging evidence (2021) suggests that public health messaging associated with COVID-19 and increased reliance on videoconferencing technologies have had a negative impact on body image [22, 23]. A recent (March 2022) mixed-studies age-inclusive systematic review of the experiences of body image, DE, and ED during the pandemic by Schneider and colleagues [24] reported that, overall, studies have shown increased shape and weight concerns [25], drive for thinness/muscularity [26], and decreased self-esteem [27].

Research specifically conducted on adolescents and body image in May 2020 found a significant association between the frequency of following appearance-focused accounts on social media (Instagram) and an increase in body and appearance dissatisfaction [28]. Another study conducted August–December 2020 by Lessard and Puhl reaffirmed that COVID-19 increases in body dissatisfaction were prevalent, with the majority of participants reporting increased exposure to weight stigmatizing social media content during the pandemic [29].

In an early pandemic mixed methods study (conducted April–May 2020, published in 2021), Buckley and colleagues collected data on body image transitions of current and former adult athletes. They noted that 34.8% (n = 69) of participants self-reported worsened body images, which were directly attributed to COVID-19 (increased body preoccupation, and fear of body composition changes) [8]. They reaffirmed that body image is one component of athletic identity; the strength with which people identify with, and embrace, their role as an athlete, including the aesthetic body image of their sport [30]. When there are changes (e.g., COVID-19 athletic disruptions, decrease in physical fitness, increase in weight), athletes can experience a sense of identity loss that increases the risk for depression [31].

To the best of these authors’ knowledge, research specifically on COVID-19 adolescent athlete changes in body image has yet to be conducted. However, based upon the above noted studies [20–28, 30–32], we can speculate that there will be a worsening of athletes’ body image over the duration of the pandemic.

### COVID-19 exacerbation of ED behavioral symptoms

Research on the exacerbations of ED during the pandemic have included initial reports (April 2020) and systematic reviews (2021-to date) among adult and adolescent populations. Touyz and colleagues provided one of the first age-inclusive perspectives on this topic (April 2020) [33]. They noted that, for many individuals with AN, lack of access to the usual forms of exercise (e.g., going to the gym) heightened concerns about gaining weight, leading to further restriction and emaciation, which increased their potential for experiencing more serious COVID-19 complications [33]. Similarly, for many individuals with BN, increased access to food while in quarantine at home, without the normal athletic exercise routine to balance out the calorie intake, resulted in reported weight gain that they feared may hinder their current and future athletic performance [33].

Buckley et al., in their study of current and former adult athletes, reported a surge in DE, including 21.1% of participants with a diagnosable ED, and 32.8% with self-reported worsened food relationships during this early phase of the pandemic [8]. Qualitative analysis indicated that DE occurred in the form of inhibitory food control, and binge eating [8]. In a subsequent scoping review of the COVID-19 ED research (November 2021), Linardon et al. reported that, in the 70 (mostly adult) selected studies, those most susceptible to pandemic symptom escalation were individuals with confirmed ED, as well as those in at-risk populations, including athletes [34].

The early impact of COVID-19 on adolescents with ED indicated increases in symptoms, impairment, and need for immediate treatment. Graell et al. reported higher reactivation of ED symptoms in adolescents compared to children from March–May 2020 [35]. During a similar time period (April–October 2020), Spettigue et al. compared a cohort of adolescent participants who presented for ED assessment before the pandemic (2019) with those who presented during the pandemic (2020) [36]. They found that pandemic cohort trended towards having lower percentage of goal weights, as well as higher rates of self-reported impairment, and were significantly more likely to be medically unstable (*p* = 0.005), and to require hospitalization (*p* = 0.005) [36]. Higher rates of inpatient admissions, emergency room consultation requests, and outpatient referrals deemed “urgent” were likewise associated with the pandemic period [36]. A very recent meta-analysis conducted by Haghshomar et al. (April 2022) focused on 13 studies with age ranges of 13–70, indicating that the overall pooled prevalence of exacerbation of binge eating, food restriction, purging behaviors, and concerns about food intake, in the sample of 7848 was 59.65% (95% CI: 49.30%; 69.60%) [37].

Similar to the body image research noted above, to the best of these authors’ knowledge, studies specifically on COVID-19 adolescent athlete exacerbation of ED behavioral symptoms have yet to be conducted. Nevertheless, the existing literature [34–38] suggests a worsening of their symptoms as the pandemic continues.

### Sport-specific pressures

Another aspect of an athlete’s identity includes how they respond to the unique pressures of their specific sport. Petrie and Greenleaf formulated a theoretical etiological model of eight mediating factors in the development of DE in athletes [39, 40]. One factor is the type of sport (individual vs. team sports, and what they termed as “lean vs. non-lean sports”**).** They note that individual sports tend to be ones that emphasize “leanness” [41], with the belief that lower body weight improves performance (e.g., track and field, martial arts) [41]. Alternatively, team sports tend to be ones that are “non-lean” [42], in that they do not require a low body weight in order for an athlete to be competitive (e.g., basketball, soccer, volleyball) [42]. “Lean” sports may increase risk for DE, in part through a syndrome known as Relative Energy Deficiency in Sport (RED-S) [43]. This involves impaired physiological function including, but not limited to, metabolic rate, menstrual function, bone health, immunity, protein synthesis, and cardiovascular health, caused by relative energy deficiency, which can affect male as well as female athletes. A recent systematic review [44] of the prevalence of DE in athletes categorized by emphasis on “leanness” and activity reported that six out of the seven included studies found a significant increase in DE rates among “lean” sport types, compared to “non-lean” sport types [44].

Sports can be further divided into six subgroups: aesthetic, weight-dependent, endurance, ball game, power, and technical sports [45]. Of these categories, aesthetic, weight-dependent, and endurance sports are typically considered “lean” sports (as defined by Petrie and Greenleaf, [39, 40]), whereas ball game, power, and technical sports are considered “non-lean” sports [39, 40]. In particular, aesthetic sports, which are judged by a complex set of rules favoring appearance (e.g., ballet, gymnastics), have been shown in sport-specific studies to have a higher prevalence of DE and ED compared to other sport subgroups [46–48]. Weight-dependent sports, which divide competitors into different categories based on their weight (e.g., wrestling, martial arts), have similarly been shown to have a higher ED pathology than those in other sport subgroups [49, 50]. Endurance sports typically associate lower body weight with a higher level of competition (e.g., track and field-running, cross-country skiing) [51].

Another sport-specific factor to consider is peer competition, which can exacerbate DE, ED, and distorted body image [52]. While both team and individual sports require competition, cooperation is much more prevalent in team than in individual sports. Thus, it is possible that team athletes’ additional goal of cooperation more evenly balances out the goal of competition, compared to individual athletes’ sole requirement for competition [53]. Additionally, peer competition can lead athletes to deny the seriousness of their concerns, and if they do seek help from a doctor, it is more likely due to complaints of decreased performance, rather than the symptoms of an ED, per se [53].

### Gender-specific pressures

Although EDs are often thought of as feminine illnesses, epidemiological studies indicate that males are also at-risk for developing ED [54–56]. Most notably, adolescent males may be less likely to seek treatment than females due to an overall higher degree of shame and stigma related to their ED [57–59]. Further, physicians and other health care providers may be less likely to recognize DE symptoms in adolescent males due differences in symptom presentation (e.g., male focus on “leanness,” vs. female focus on weight) [58, 59].

These gender-specific pressures can overlap with the above-noted sport-specific pressures. A systematic review [60] of risk factors for eating psychopathology included a study which examined the relationship between gender, type of sport (“lean” vs. “non-lean”), body dissatisfaction, and self-esteem, with DE behaviors in Division 1college athletes [60]. They found that participating in “lean” sports was associated with increased DE and body dissatisfaction for male athletes, but not female athletes [61]. This unique vulnerability for male athletes, in addition to the factors related to lower likelihood of treatment for males (e.g., stigma, shame, provider misconceptions), suggests that male athletes should be targeted by sport psychologists, coaches, and other sports professionals, for prevention and early intervention for DE, ED, and distorted body image [62].

### Aim of the present study

COVID-19 thus presents specialized issues for adolescent athletes, particularly those at risk for, or experiencing, DE, ED, and associated distorted body image [3, 5–12]. Specific pressures of individual sports (which tend to emphasize “leanness” as a means to improving performance), versus team sports (which tend to not require “leanness” for an athlete to be competitive), may exacerbate these concerns. An additional factor to consider is the gender of the athletes, with participation in “lean” sports associated with increased DE and body dissatisfaction for male, but not female, athletes. The aim of the present study was therefore to compare ED symptoms and body image between individual and team sport adolescent male athletes living in one of the most affected areas of Iran at the beginning of COVID-19.

## Methods

### Participants

Participants of the study included 124 Iranian male adolescent athletes residing in the Mazandaran province (one of the most affected areas of Iran during COVID-19), who were recruited through the three University study sites nearby (Farhangian University, Tarbiat Modares University, and Islamic Azad University). The athletes played in 1 of 6 sports (individual or group): (1) Taekwondo (individual); (2) Track and Field (individual); (3) Wrestling (individual); (4) Volleyball (group); (5) Basketball (group); or (6) Soccer (group).

Study inclusion criteria were: (1) aged 12 to 19 years old (“adolescence,” as defined by Rice, F.P., in Human Development: A Life-Span Approach) [63]; (2) completion of a physical illness history measure, assessed pre-study through participant self-report via the WHO Global Physical Activity Questionnaire (WHO-GPAQ) [64] (no disabilities or physical problems were reported by the research participants); (3) completion of a mental illness history measure, assessed pre-study through participant self-report via the WHO-Composite International Diagnostic Interview (WHO-CIDI) [65] (no specific, diagnosable mental illnesses were reported by the research participants); (4) sports history for at least 3 years, assessed via the WHO-GPAQ; and (5) written consent by the adolescents and their parents for participation in the research. The study exclusion criterion was: (1) lack of regular physical activity, assessed via the WHO-GPAQ. This criterion was included in order to differentiate between a lack of regular physical activity due to normal patterns (e.g., sedentary lifestyle), versus a lack of regular physical activity due to changes during the COVID-19 pandemic assessment period (e.g., reduced exercise levels). Of note, all included participants had continued exercise during the pandemic (see details below). The amount of regular physical activity was tracked by both the adolescent athletes and their parents, and the accuracy of the level of activity was confirmed by the study researchers. Thus, all participant responses were equal in terms of the associations between activity and scores.

Of the eligible participants who were available during a COVID-19 quarantine period from June through August 2020 (N = 180), the Krejcie and Morgan Sampling Method was used to simplify the process of determining the sample size for a finite population [66], resulting in a calculation of N = 124 sample participants. In order to maximize statistical power, the eligible participants were then selected to be divided equally into two groups based upon their sport affiliation: the individual sport group (n = 62), and the team sport group (n = 62). Throughout the data collection quarantine time, all athletes practiced and played at home, or in socially isolated environments, depending on the training that the coaches designed for them. Thus, the athletes had continued exercise during the lockdown, albeit with modified routines and formats.

### Procedure

The governing Medical Ethics and History of Medicine Research Center for each Iranian university study site reviewed and approved the study before enrollment. Participants were recruited via a study research coordinator who had contacted the respective officials of the 6 sports groups. The study procedures were explained, and informed consent was obtained from all participants and their parents prior to study initiation.

### Measures

De-identified study data (sociodemographic variables, questionnaires) were collected online at the participants’ homes during the quarantine period of COVID-19. The Persian (Farsi) translations of the questionnaires were used for the participants’ comfort and familiarity with the language. These translated versions were evaluated, and found to be reliable and valid for the Iranian population (please see below for the psychometrics of each questionnaire).

### WHO-GPAQ

The WHO-GPAQ collects information on physical activity participation in three domains (activity at work, travel to and from places, recreational activities), as well as sedentary behavior, defined as “any waking behavior characterized by a low energy expenditure (e.g., sitting, as with desk work, driving a car, watching television)” [64]. Participants completed the self-report version pre-study to denote their histories of physical illness and sports participation. In a nine country reliability and validity study of the WHO-GPAQ, Bull and colleagues reported that the inter-rater and test–retest reliability coefficients were of moderate to substantial strength (Kappa *k* = 0.67–0.73; Spearman's rho ρ = 0.67–0.81, *p* < 0.01, respectively) [67]. Results on concurrent validity between the WHO-GPAQ and the International Physical Activity Questionnaire (IPAQ), a previously validated and accepted measure of physical activity [68], also showed a moderate to strong positive relationship (Pearson’s product-moment correlation coefficient *r* = 0.45–0.65) [67]. In the present study, the Persian (Farsi) translation version of the whole scale WHO-GPAQ had an internal consistency reliability Cronbach's alpha α = 0.93 [69]. Utilizing Lawshe’s technique to assess content validity [70], subject matter experts review each measurement question in order to determine its individual content validity ratio (CVR), with a final mean CVR of all of the questions denoted as the content validity index (CVI) (the closer the CVI is to 1, the higher the overall content validity of the measure). In this translation version, the CVI = 0.72 [69].

### WHO-CIDI

The WHO-CIDI includes a screening module and 41 sections that focus on mental health diagnoses, functioning, treatment, risk factors, socio‐demographic correlates, and different methodological factors [65]. The WHO-CIDI generates diagnoses of mental disorders according to the criteria of the DSM-5 [18]. Participants completed the self-report version pre-study to denote their history of mental illness. In a study of the reliability, validity, and factorial structure of the WHO-CIDI in Iranian psychiatric outpatients, Dadfar and Kalibatseva reported an internal consistency reliability Cronbach’s alpha α = 0.91 [71]. The WHO-CIDI negatively correlated with the Patient Health Questionnaire-9 (PHQ-9) [72] (Pearson’s product moment correlation coefficient *r* =  − 0.358), Patient Health Questionnaire-15 (PHQ-15) [73] (Pearson’s product moment correlation coefficient *r* =  − 0.328), and the Beck Depression Inventory-13 (BDI-13) [74] (Pearson’s product moment correlation coefficient *r* =  − 0.475), indicating good validity [71]. In the present study, the Persian (Farsi) translation version of the whole scale WHO-CIDI had an internal consistency reliability Cronbach's alpha α = 0.83, and CVI = 0.72 [75].

### Eating attitudes test-26 (EAT-26)

The EAT-26 [76] is a 26-item self-report measure with 3 subscales: (1) thoughts and behaviors related to dieting (Dieting); (2) preoccupation with food and impulses to binge and purge (Bulimia and Food Preoccupation); and (3) attempts to control food intake (Oral Control). Response agreement is rated on a 4-point Likert scale (0 = “Never,” to 3 = “Always”), with total scores ranging from 0 to 78 (clinical cut-off score of ≥ 20 indicates disordered eating). Garner and colleagues reported that the wholescale EAT-26 score items have a “good” [77] internal consistency reliability Cronbach’s alpha α = 0.88, and the Dieting subscale items have an “excellent” [77] internal consistency reliability Cronbach’s alpha α = 0.90. While the other two internal consistency reliabilities were not initially reported, a subsequent meta analyses of the EAT-26 literature by Gleaves et al. [78] reported a mean “questionable” [77] internal consistency reliability Cronbach’s alpha α = 0.67 for the Bulimia and Food Preoccupation subscale, and a mean “poor” [77] Cronbach’s alpha α = 0.56 for the Oral Control subscale. However, the wholescale EAT-26 score items have a content validity Pearson’s product moment correlation *r* = 0.91, and the 3 subscales have a content validity Pearson’s product moment correlation *r* = 0.78 [76].

Research findings have indicated that the wholescale EAT-26 score items in general populations and patient samples have been shown to be highly reliable (internal consistency reliability Cronbach’s alpha α = 0.91; test–retest reliability Spearman’s rho ρ = 0.98, *p* < 0.01), and valid (positively correlated with the Structured Clinical Interview for Axis I DSM-IV Disorders (SCID) for Module H [79]; criterion validity Pearson’s product moment correlation *r* = 0.90) [80–82]. In the present study, the Persian (Farsi) translation version of the wholescale EAT-26 had an internal consistency reliability Cronbach's alpha α = 0.90, and the test–retest reliability for the EAT-26 3 subscales was good (Spearman’s rho ρ = 0.84–0.89, *p* < 0.001) [83].

### Body-esteem scale for adolescents and adults (BESAA)

The BESAA [84] is a 23-item self-report measure with 3 subscales: (1) feelings regarding one’s appearance (Appearance); (2) perceptions of others’ evaluations about one’s appearance (Attribution); and (3) weight satisfaction (Weight). Response agreement is rated on a 5-point Likert scale (1 = “Never,” to 5 = “Always”), with total scores ranging from 23 to 115 (higher scores indicate more positive body-esteem). Mendelson and colleagues reported that the BESAA items have an internal consistency reliability Chronbach’s alpha α = 0.89, Spearman’s rho ρ = 0.88, *p* < 0.01, and a content validity Pearson’s product moment correlation *r* = 0.72 [84]. In the present study, the Persian (Farsi) translation version of the BESSA demonstrated good model fit statistics (χ^2^/df = 3.41, *p* < 0.001), and good internal consistency reliability for the 3 subscales: (1) BESSA-Appearance (Chronbach’s alpha α = 0.76), (2) BESSA-Attribution (Chronbach’s alpha α = 0.69), and (3) BESSA-Weight (internal consistency reliability Chronbach’s alpha α = 0.85) [85]. The test–retest reliability for the BESSA 3 subscales was moderate (Spearman’s rho ρ = 0.57–0.68, *p* < 0.01) [85].

### Data analyses

All data were entered into the Statistical Package for Social Science (SPSS) 24 software for analyses [86]. Sociodemographic variables were collected for the individual and team athlete groups. The Kolmogorov–Smirnov Test [87] was used to check the normality of the EAT-26 and BESSA data (normality is the first statistical assumption required for the subsequent Multiple Analysis of Variance (MANOVA)) [88]. Of note, MANOVA was chosen instead of a single *t*-test with multiple dependent variables in order to limit the joint error rate. The M-Box Test of Equality of Covariance Matrices [89] was used to check for the equality of covariance matrices for the overall differences in EAT-26 and BESSA scores between the individual and team athlete groups (equality of covariance matrices is the second statistical assumption required for the subsequent MANOVA). Levene’s Test of Homogeneity of Variance [90] was used to check the homogeneity of variance for the EAT-26 and BESSA specific subscale scores of the individual and team athlete groups (homogeneity of variance is the third statistical assumption required for the subsequent MANOVA). With the above statistical assumptions being met, the MANOVA detailed the specific dependent variables (individual EAT-26 and BESSA subscale scores) that were significantly different between the individual and team athlete groups.

## Results

### Individual and team athlete groups sociodemographic variables

The sociodemographic variables of the individual (*n* = 62) and team (*n* = 62) athlete groups are presented in Table 1. In summary, individual athletes had significantly higher mean values of age, height, weight, and Body Mass Index (BMI; as calculated by the Centers for Disease Control and Prevention (CDC) BMI Percentile Calculator for Child and Teen [91]) compared to team athletes (*p* = 0.001). However, the range of distribution for each of these variables is a tight cluster of scores, as detailed below. Individual athletes had a significantly higher mean age of 14.1 years (Standard Deviation, *SD* = 0.6 years, range = 12.2–18.4 years) compared to team athletes (mean = 13.9 years, *SD* = 0.8 years, range = 12.1–18.7 years) (*p* = 0.001). Individual athletes had a significantly higher mean height of 157.2 cm (*SD* = 7.2 cm, range = 154.1–182.5 cm) compared to team athletes (mean = 156.7 cm, *SD* = 7.6 cm, range = 149.1–164.3 cm) (*p* = 0.001). Individual athletes had a significantly higher mean weight of 66.1 kg (*SD* = 1.6 kg, range = 64.3–87.1 kg) compared to team athletes (mean = 64.7 kg, *SD* = 1.8 kg, range = 64.0–75.1 kg) (*p* = 0.001). Individual athletes had a significantly higher mean Body Mass Index (BMI) of 21.1 (*SD* = 2.1, range = 20.3–22.4) compared to team athletes (mean = 20.1, *SD* = 2.2, range = 19.8–22.1) (*p* = 0.001).

**Table 1:** Sociodemographic variables of the individual and team athlete groups

Variable	Individual athlete group (n = 62)		Team athlete group (n = 62)			
	Mean	SD	Mean	SD	T	*p*-value
Age	14.1	0.6	13.9	0.8	6.9	0.001
Height (cm)	157.2	7.2	156.7	7.6	5.7	0.001
Weight (kg)	66.1	1.6	64.7	1.8	3.1	0.001
BMI	21.1	2.1	20.1	2.2	2.8	0.001

SD = Standard Deviation; cm = centimeters; kg = kilograms; BMI = Body Mass Index, as calculated by the Center for Disease Control and Prevention (CDC) BMI Percentile Calculator for Child and Teen [91]

### Statistical tests for the assumptions of the overall MANOVA

The M-Box Test of Equality of Covariance Matrices [89] for the overall differences in EAT-26 and BESSA scores between the individual and team athlete groups is shown in Additional file 1: Table S1. Levene’s Test of Homogeneity of Variance [90] for the homogeneity of variance in the EAT-26 and BESSA subscale scores between the individual and team athlete groups is presented in Additional file 1: Table S2. The MANOVA for the overall differences in EAT-26 and BESSA scores of the individual and team athlete groups is noted in Additional file 1: Table S3. In summary, 49.7% of the variance related to the difference between the individual and team athlete groups is due to the interaction of all of the dependent variables.

### MANOVA specific dependent variables

Table 2 details the specific dependent variables (individual EAT-26 and BESSA subscale scores) in the MANOVA that had significant differences between the individual and team athlete groups. In summary, the individual athlete group had significantly higher scores on the EAT-26 Bulimia and Food Preoccupation subscales (*p* = 0.019), and significantly higher scores on the BESSA Appearance (*p* = 0.001), Weight (*p* = 0.001), and Attribution (*p* = 0.001) subscales, compared to the team athlete group. However, there were no significant differences between the two athlete groups on the EAT-26 Dieting and Oral Control subscales.

**Table 2:** MANOVA for the specific difference in EAT-26 and BESSA subscale scores of the individual and team athlete groups

Dispersion source	Variable	Sum of squares	*df*	Average of squares	F	*p*-value
Group	EAT-26 dieting	8.002	1	8.002	1.102	0.296
	EAT-26 oral control	6.099	1	6.099	0.697	0.405
	EAT-26 bulimia and food preoccupation	14.842	1	14.842	5.666	0.019
	BESSA appearance	72.434	1	72.434	30.278	0.001
	BESSA weight	61.577	1	61.77	23.181	0.001
	BESSA attribution	19.828	1	19.828	24.599	0.001

MANOVA = Multiple analysis of variance [88]; EAT-26 = Eating attitudes test-26 [76]; BESSA = Body-esteem scale for adolescents and adults [84]

## Discussion

The aim of the present study was to compare ED symptoms and body image between individual and team sport adolescent male athletes who were geographically located in one of the most affected areas of Iran during the beginning of COVID-19. In summary, the individual athlete group had significantly higher scores compared to the team athlete group on subscales focused on preoccupation with food and impulses to binge and purge, feelings regarding one’s appearance, perceptions of others’ evaluations about one’s appearance, and weight satisfaction. However, there were no significant differences between the two athlete groups on subscales focused on thoughts and behaviors related to dieting, and attempts to control food intake.

As noted above, while research on the specific population of individual and team sport male adolescent athletes with exacerbated eating and body image distortions during COVID-19 is very limited, given the narrow focus of this study group, and the acute onset of the pandemic, we can draw from the available literature for comparisons with the present study. Overall, our results of significant differences between the individual and team athlete groups on subscale scores is consistent with the majority of the research that was conducted pre-COVID-19 [54–56, 92].

A relatively early study by Rodriguez et al. (1999) reported that DE symptoms (e.g., diets, compulsive eating, and compensatory behaviors) were more frequent in adolescent exercisers who participated in individual sports compared with those participating in team sports [93]. Subsequent research by Milligan et al. [61] found that participating in “lean” sports was associated with increased DE and body dissatisfaction for university male athletes, but not female athletes. A 2018 review of ED in adolescent and young adult males by Limbers and colleagues indicated that athletes who participate in sports that value “leanness” (e.g., most individual sports) may experience pressure from coaches and/or teammates to engage in unhealthy weight control practices [62]. A systematic review by Stoyel et al. of risk factors for eating psychopathology in 2019, including articles published from 2000 to 2018, examined studies with EAT-26 scores [60]. They highlighted a finding from Kong and Harris [42] on adolescent and adult (age range 17–30 years) female athletes, where those who participated in “lean” sports had an increased rate of self-induced vomiting and laxative use, compared with the “non-lean” sport participants. In a study by Pamuk and colleagues on eating attitudes in 502 male and female university athletes who completed the EAT-26, conducted and published just before COVID-19 (2019-early 2020), individual athletes were found to have significantly higher wholescale scores, and on each of the Dieting, Bulimia and Food Preoccupation, and Oral Control subscale scores, compared to team athletes (*p* < 0.05) [92]. This was the case when analyzing the entire sample (both genders), as well as when male athletes only were selected in a subsample [92]. Mancine et al. [44] conducted a systematic review on the prevalence of DE in athletes categorized by emphasis on “leanness” and activity type (included articles were from January 2000–July 2019, and the findings were published in 2020). They reported that 6 out of the 7 included studies (males and females, age range 12–30 years old) found a significant increase in DE rates among “lean” sport types [44]. A population-based study of DE in 10,172 adolescents participating in individual and team sports by Heradstveit et al. [94] (data collected in 2012, research published in 2020) noted that DE symptoms were associated with higher individual sports participation compared to those participating in team sports. Pre-COVID-19 research thus tends to highlight the significant DE differences between individual (mostly “lean”) and team (mostly “non-lean”) athletes [94, 95].

In the current study, our findings of individual athlete increased scores on the EAT-26 Bulimia and Food Preoccupation, and BESSA Appearance, Weight, and Attribution, subscales compared to team athletes are in line with the above noted research on individual, “lean” sports. Indeed, individual athlete pressure to maintain a “lean” body for their sport by any means necessary can include the behavior of purging (bulimia) [44]. In addition, dietary restriction has been shown to lead to an increase in food preoccupation (anorexia) [44]. Further, an individual athlete’s identity tends to be tied more to their appearance, weight, and attribution, compared to a team athlete’s identity [30].

Our results indicating no differences between the individual and team athlete groups on the EAT-26 Dieting and Oral Control subscales, however, is consistent more with emerging research on the effects of COVID-19 and exacerbation of DE, ED, and body image distortion. Buckley et al., in their study of current and former adult athletes conducted during the early transition period of the pandemic in April–May 2020, reported a surge in DE in all participants (N = 162; male and female athletes, from a range of 41 different individual and team sports) [8]. This included 21.1% of participants scoring above the EAT-26 20 point cut-off, suggesting an ED [76], 34.8% of participants self-reporting worsened body image, and 32.8% self-reporting a worsened food relationship, directly attributed to COVID-19 [8]. Qualitative analysis indicated that DE occurred predominantly in the form of body preoccupation, inhibitory food control, fear of body composition changes, and binge eating [8]. Interestingly, they found no significant differences between the individual and team sport groups, type of sporting category (endurance, antigravitational, ball sport, power, technical and aesthetic), or level of competition (club, state, national or international) [8]. Denerel et al. [5], in their subsequent study on the effects of long-duration home isolation linked to the pandemic on the mental health of adolescent athletes (June 2021), reported a decrease in physical activity (88%) and an increase in depression (42.8%) in their participants (n = 940 team athletes, n = 274 individual athletes, n = 131 nonathlete controls, boys and girls, age range 12–17 years). Posttraumatic stress symptoms were lower among athletes than nonathlete controls for girls, but similar for boys [5]. However, they found no significant differences in depression, state, and trait anxiety symptoms between the individual and team athletes [5].

The relationship between dieting (behaviors and thoughts related to dieting) and oral control (attempts to control food intake) tends to become a vicious, reinforcing cycle for many people. Behavioral attempts to control food in turn solidify cognitive control over food, which increases food preoccupation [8]. Inhibitory control has a role in maintaining DE, including restrict-binge cycles, and more specifically in athletes, it can function to enhance performance focus (including areas where they feel that they have failed to meet their/others’ expectations) [96]. This interaction between behavioral and cognitive control may further modulate stress in such an uncertain transition as the COVID-19 pandemic [8]. In order to address the clinical implications of these findings for sport psychologists, coaches, and other sports professionals working with both individual and team athletes, Buckley et al. [8] suggest some key Practice Principles that utilize athlete descriptions of improvements in their food-body relationship over a transition. Principles related to dieting and oral control include: (1) Control (channeling control outside of the body, and away from restrictive dietary practices, or rigid body controlling movement); (2) Variety (keeping a range of food and exercise open to counteract restraint and cognitive inflexibility); and (3) Adaptability (finding regularity in diet practices where possible, but ultimately being able to adapt to altered appetite needs, food environment changes, and food access). Thus, sports professionals may help individual and team athletes improve these skills.

Buckley and colleagues recommend additional Practice Principles that have been shown in previous studies to mend the relationships between athletes, their eating practices, and their body images [8]. These include: (1) Support (engaging with specialized options to navigate potential DE development, body image concerns, mental health challenges, and social comparison); (2) Connection (connecting to one’s changing needs through intuitive eating or intuitive movement, and being able to adapt to the body’s physical needs related to training and exercise), and (3) Acceptance (accepting that the body is not infinitely malleable, and seeking acceptance with non-judgement through body shape, weight, and composition changes) [8].

Research on DE, ED, and body image distortion in male adolescent athletes during COVID-19 is in its relative infancy. Findings thus far have indicated that a large number of athletes have experienced a worsening of their symptoms during the pandemic, highlighting the importance of providing specialized support towards reducing body related anxiety, vicious food control cycles, and maladaptive exercise management, during such times of transition. Further longitudinal research is needed in order to determine the long-term effects on this population. Overall, these findings underscore the need for sport psychologists, coaches, and other sports professionals working with male adolescent athletes (individual and team), to monitor DE, ED, and distorted body image during COVID-19, in order to provide early intervention, and mitigate the risk of long-term consequences.

### Clinical implications

In the current study, there were several important findings and clinical implications related to the athletic settings that the participants were in (individual vs. team sports). As noted above, COVID-19 presents specialized issues for adolescent athletes, particularly those at risk for, or experiencing, DE, ED, and distorted body image [3, 5–12]. While individual athletes had significantly higher scores than team athletes on most EAT-26 and BESSA subscales, there were no differences between the groups on the EAT-26 subscales of Dieting and Oral Control. The specific relationship between dieting (behaviors and thoughts related to dieting) and oral control (attempts to control food intake) tends to become a vicious, reinforcing cycle for many people. Improvement of skills related to control (channeling away from restrictive dietary practices), variety (keeping a range of food open to counteract restraint and cognitive inflexibility), and adaptability (being able to change with altered appetite needs, food environment, and access) may improve this dieting-oral control relationship among athletes. Additionally, skills related to support (engaging with specialized options to navigate DE development), connection (to one’s changing needs through intuitive eating or intuitive movement), and acceptance (non-judgement of body shape, weight, and composition changes) may mend the relationships between athletes, their eating practices, and their body images. Overall, the findings highlight the need for sport psychologists, coaches, and other sports professionals working with male adolescent athletes (individual and team), to monitor DE, ED, and distorted body image during COVID-19, in order to provide early intervention, and mitigate the risk of long-term consequences.

### Limitations

The findings of the current study must be viewed within the context of certain limitations.

First, the study utilized a sample of Iranian male adolescent athletes during the beginning of COVID-19 quarantine (June–August 2020); therefore, results cannot be generalized to other populations. Future research should endeavor to conduct similar studies in other populations, in order to compare, and potentially replicate, the results. Second, as noted above, research on the specific population of adolescent male athletes with existing/exacerbating DE/ED symptoms and body image disturbance during the pandemic is very limited given the narrow focus of this group, and the acute onset of the pandemic. However, we have attempted to draw from the existing studies on aspects of this population and dependent variables of interest in order to compare and contrast findings in the literature, and hopefully contribute to research on this particular high-risk population. Third, due to the early assessment period for this study, structured measurements of pandemic barriers (e.g., impact on physical and mental health for individuals, family, and friends) had not yet been developed. As these scales are now available, they should be utilized in conjunction with other instruments of interest. Fourth, the participant self-reports were not supported by additional investigator conducted diagnostic interviews, which could lead to underreporting of these concerns. Subsequent research should endeavor to also include investigator interviews (e.g., by tele-interview during COVID-19) to support any self-report measures. Fifth, we did not conduct sport-specific analyses due to concerns of low statistical power. It is possible that the characteristics of the athletes within one or two sports may be responsible for the differences that were seen between groups, and the conclusions might thus be more specific than we presented. Future research could increase the overall and sport-specific subgroup samples in order to provide additional cell sizes for more robust analyses. Finally, the cross-sectional assessment of ED and body image in the current study preclude causal inferences for these relationships. Prospective research could employ a more experimental, longitudinal design, in order to elucidate the effects of each of these variables.

## Conclusions

COVID-19 presents specialized issues for adolescent athletes, particularly those at risk for, or experiencing, DE, ED, and distorted body image [3, 5–12]. In summary, in the current study, while individual athletes had significantly higher scores than team athletes on most EAT-26 and BESSA subscales, there were no differences between the two groups on the EAT-26 subscales of Dieting and Oral Control. Clinical implications for these findings include the improvement of skills related to control, variety, adaptability, support, connection, and acceptance, which may improve the relationships between athletes, their eating practices, and their body images) [8]. Overall, the findings highlight the need for sport psychologists, coaches, and other sports professionals working with male adolescent athletes (individual and team), to monitor DE, ED, and distorted body image during COVID-19, in order to provide early intervention, and mitigate the risk of long-term consequences.

## Supplementary Information

**Additional file 1: Table S1.** M-Box test of equality of covariance matrices for the overall difference in EAT-26 and BESSA scores of the individual and team athlete groups. **Table S2.** Levene’s test of homogeneity of variance for the EAT-26 and BESSA subscale scores of the individual and team athlete groups. **Table S3.** MANOVA for the overall difference in EAT-26 and BESSA scores of the individual and team athlete groups

## Data Availability

The datasets generated and analyzed during the current study are not publicly available, as individual privacy could be compromised, but are available from the corresponding author on reasonable request.

## Abbreviations

AN: Anorexia nervosa
BESAA: Body-esteem scale for adolescents and adults
BMI: Body mass index
BN: Bulimia nervosa
CDC: Center for disease control and prevention
cm: Centimeters
CVI: Content validity index
CVR: Content validity ratio
DSM-5: Diagnostic and statistical manual of mental disorders-5
EAT-26: Eating attitudes test-26
EBT: Evidence based treatment
ED: Eating disorders
IPAQ: International physical activity questionnaire
kg: Kilograms
MANOVA: Multiple analysis of variance
SD: Standard deviation
SPSS: Statistical package for social science
WHO: World Health Organization
WHO-CIDI: WHO-composite international diagnostic interview
WHO-GPAQ: WHO global physical activity questionnaire
