# Supplementary material for: Comparison of eating disorders symptoms and body image between individual and team sport adolescent athletes during the COVID-19 pandemic
Source: J Eat Disord. 2022 Aug 12;10:119. doi: 10.1186/s40337-022-00644-4 (PMC9372990; doi:10.1186/s40337-022-00644-4)
Supplement: Supplementary file 1 — Additional file 1: Table S1. M-Box test of equality of covariance matrices for the overall difference in EAT-26 and BESSA scores of the individual and team athlete groups. Table S2. Levene’s test of homogeneity of variance for the EAT-26 and BESSA subscale scores of the individual and team athlete groups. Table S3. MANOVA for the overall difference in EAT-26 and BESSA scores of the individual and team athlete groups [file 40337_2022_644_MOESM1_ESM.docx]

**Supplemental Table 1. M-Box Test of Equality of Covariance Matrices for the Overall Difference in EAT-26 and BESSA Scores of the Individual and Team Athlete Groups**

| **M-Box** | **39(941)** |
| --- | --- |
| F | 1.563 |
| df1 | 1(21) |
| df2 | 45130(932) |
| p | 0.287 |

M-Box Test of Equality of Covariance Matrices (24); EAT-26 = Eating Attitudes Test- 26 (19); BESSA = Body-Esteem Scale for Adolescents and Adults (20).

The M-Box Test of Equality of Covariance Matrices tests the null hypothesis that the variance–covariance matrices are the same in both groups. Therefore, if the matrices are equal (and thus the assumption of homogeneity is met) this statistic should be *non-significant.* The test is in fact non-significant (*F*(1, 21) = 1.56, *p* = .287), indicating the matrices can most likely be homogenized.

**Supplemental Table 2. Levene’s Test of Homogeneity of Variance for the EAT-26 and BESSA Subscale Scores of the Individual and Team Athlete Groups**

| **p** | **dF2** | **df1** | **F** | **Variable** |
| --- | --- | --- | --- | --- |
| 0.188 | 122 | 1 | 2.466 | EAT-26  Dieting |
| 0.210 | 122 | 1 | 1.588 | EAT-26  Oral Control |
| 0.268 | 122 | 1 | 1.236 | EAT-26  Bulimia and Food Preoccupation |
| 0.585 | 122 | 1 | 0.300 | BESSA  Appearance |
| 0.246 | 122 | 1 | 1.360 | BESSA  Weight |
| 0.117 | 122 | 1 | 2.466 | BESSA  Attribution |

Levene’s Test of Homogeneity of Variance (25); EAT-26 = Eating Attitudes Test- 26 (19); BESSA = Body-Esteem Scale for Adolescents and Adults (20).

Levene’s Test of Homogeneity of Variance should be non-significant for all dependent variables if the assumption of homogeneity of variance has been met. Each of the subscale tests are non-significant : EAT-26 Dieting subscale (*F*(1,21) = 2.47, *p* = .188); EAT-26 Oral Control subscale (*F*(1,21) = 1.59, *p* = .210); EAT-26 Bulimia and Food Preoccupation subscale (*F*(1,21) = 1.24, *p* = .268); BESSA Appearance subscale (*F*(1,21) = 0.30, *p* = .585); BESSA Weight subscale (*F*(1,21) = 1.36, *p* = .246); and BESSA Attribution subscale (*F*(1,21) = 2.47, *p* = .117).

**Supplemental Table 3. MANOVA for the Overall Difference in EAT-26 and BESSA Scores of the Individual and Team Athlete Groups**

| **Effect** |  | **Value** | **F** | **Hypothesis df** | **Error df** | **p** | **Partial Eta Squared** |
| --- | --- | --- | --- | --- | --- | --- | --- |
| Group | Pillai's Trace | 0.497 | 93.42 | 2 | 121 | **.001** | .497 |
|  | Wilks’ Lambda | 0.503 | 93.42 | 2 | 121 | **.001** | .497 |
|  | Hotelling’s Trace | 0.987 | 93.42 | 2 | 121 | **.001** | .497 |
|  | Roy's Largest Root | 0.987 | 93.42 | 2 | 121 | **.001** | .497 |

MANOVA = Multiple Analysis of Variance (56); EAT-26 = Eating Attitudes Test- 26 (19); BESSA = Body-Esteem Scale for Adolescents and Adults (20).

The results of the overall MANOVA show that there is a significant difference between the individual and team athlete groups in all of the dependent variables (EAT-26 and BESSA subscale scores) in general (F(2, 121) = 93.42, p < .0005; Wilk's Λ = 0.503, partial η^2^ = .497). Thus, 49.7% of the variance related to the difference between the individual and team athlete groups is due to the interaction of all of the dependent variables.
